# Distinguishing specific from broad genetic associations between external correlates and common factors

**DOI:** 10.1093/bioinformatics/btaf568

**Published:** 2025-10-14

**Authors:** Javier de la Fuente, Diego Londoño-Correa, Elliot M Tucker-Drob

**Affiliations:** Department of Psychology, The University of Texas at Austin, Austin, TX 78712, United States; Department of Psychology, The University of Texas at Austin, Austin, TX 78712, United States; Department of Psychology, The University of Texas at Austin, Austin, TX 78712, United States

## Abstract

**Motivation:**

Within the genomic structural equation modelling (genomic SEM) framework, common factors are often used to index shared genetic etiology across constellations of genome-wide associations studies (GWASs) phenotypes. A standard common pathway model, in which a genetic association is estimated between an external GWAS phenotype and a common factor, assumes that all genetic associations between the external GWAS phenotype and the individual indicator phenotypes are mediated through the factor. This assumption can be tested using the Q_Trait_ statistic, which compares the common pathway model to an independent pathways model that allows for direct genetic associations between the external GWAS phenotype and the individual indicators of the factor. However, Q_Trait_ is not designed to identify either the magnitude or the source of this heterogeneity.

**Results:**

We expand upon the Q_Trait_ approach by describing an effect size index that quantifies the degree to which the common pathways model is violated, and we provide a systematic approach for empirically identifying specific direct pathways between an external trait and indicator traits. Our method comprises a series of omnibus tests and outlying indicator detection algorithms indexing the heterogeneity of associations between the genetic component of external traits and the individual indicators of common factors. We provide a set of automated functions which we apply to investigate the patterns of genetic associations across a set of external correlates with respect to indicators of general cognitive ability and case-control and proxy GWAS indices of Alzheimer’s disease.

**Availability and implementation:**

The Genomic SEM R package and the Q_Trait_ function is available at https://github.com/GenomicSEM/GenomicSEM. The Q_Trait_ function tutorial is available at https://github.com/GenomicSEM/GenomicSEM/wiki/8.-Tutorials. To ensure reproducibility of the analyses presented in this manuscript, the exact version of the Q_Trait_ function used, along with input data and scripts, has been archived on Zenodo (DOI: https://doi.org/10.5281/zenodo.17186083)

## 1 Introduction

Recent multivariate genome-wide associations studies (GWASs) have revealed shared genetic architecture across diverse constellations of biobehavioral traits. Within the genomic structural equation modeling (genomic SEM) framework (Grotzinger *et al.* 2019), common factors are often used to index and characterize shared genetic architecture at different levels of analysis. Common factors account for shard genetic etiology across multiple GWAS traits, providing a parsimonious representation of the empirical patterns of genetic covariance across the traits. In many applications, a key goal is to estimate genetic relations between these common factors and external GWAS traits. This is especially appealing because it reduces the number of genetic correlations (or genetic regression coefficients) estimated, and consolidates signal (and thus power) across indicators of a given factor (Grotzinger *et al.* 2022, Schwaba *et al.* 2025).

However, a key assumption of such a model is that the genetic associations between the external trait and the individual indicators is well-represented by associations between the external trait and the factor(s) on which the indicators load. Put differently, the standard common pathway model assumes that all genetic associations between the external GWAS correlates and the individual indicator phenotypes are mediated through the common factor. In instances in which the model fails to adequately capture the underlying pattern of associations, additional associations between the external trait and one or more individual indicators (direct effects) may be warranted. Grotzinger *et al.* (2022) introduced the Q_Trait_ statistic as an omnibus test of heterogeneity of relations between an external trait and indicator traits beyond that implied by the common pathways model. Q_Trait_ is a χ^2^ distributed test statistic that is estimated by comparing a model with pathways from the external GWAS correlate to the factor with a model in which the external correlate has direct pathways to the individual indicator phenotypes that load onto the common factor. A significant Q_Trait_ statistic indicates that the relationship between the GWAS indicator traits and the external correlate cannot be solely explained by pathways through the common factor(s), implying the existence of more specific underlying pathways. If such specific pathways are omitted from the model, the estimate of the association between the trait and the factor may be biased.

The Q_Trait_ statistic can detect heterogeneous associations between common factors and external correlates, but it is not designed to identify either the source or the magnitude of this heterogeneity. As an omnibus inferential test, it evaluates heterogeneity based on statistical significance, without accounting for effect size. Ignoring effect size may lead to the detection of trivial differences in high-powered GWAS studies, while in low-powered studies, meaningful differences might be mistaken for sampling variation. Furthermore, identifying outlying indicators can provide valuable insights into specific GWAS phenotypes that follow distinct pathways of association with external correlates. This identification is crucial, as it may reveal unique genetic pathways linking individual traits to external correlates, providing a foundation for generating testable hypotheses about the genetic mechanisms underlying complex traits.

Here, we provide a systematic approach to investigate and correct for potential heterogeneity of associations between the genetic component of external correlates and the individual indicators of common factors underlying shared genetic architecture. Our approach combines both statistical significance and effect size measures of heterogeneity (lSRMR), and provides a systematic approach for identifying the specific indicator phenotypes for which direct pathways may be added to the standard common pathways model. Moreover, we provide a set of automated functions within the genomic SEM framework and illustrate their application to investigate the patterns of associations across a set of external correlates with respect to general cognitive function and Alzheimer’s disease (AD). We used the Q_Trait_ function to explore the patterns of genetic associations across a set of external biobehavioral correlates with respect to two genetic factors representing: (i) genetic propensity toward AD using case-control GWAS of AD and proxy-phenotype GWAX of maternal and paternal history of AD and (ii) a genetic general intelligence factor (*g*) indexing shared genetic variation among seven cognitive traits. For AD, we used the genomic SEM-based multivariate model proposed by de la Fuente *et al.* (2022) to represent the overall genetic predisposition toward AD as a common factor influencing both the direct GWAS phenotype and two GWAX phenotypes. For general cognitive function we used the genetic general intelligence factor (g) described in de la Fuente *et al.* (2021).

## 2 Materials and methods

### 2.1 Overview of genomic SEM

Genomic SEM is a two-stage structural equation modeling approach. In the initial stage, a genetic covariance matrix (S) and its associated sampling covariance matrix (V) are estimated using a multivariate version of linkage disequilibrium score regression (LDSC). S consists of heritabilities on the diagonal and genetic covariances (coheritabilities) on the off-diagonal. V comprises squared standard errors of S on the diagonal and sampling covariances on the off-diagonal, which capture dependencies between estimating errors, particularly in cases where there is participant sample overlap across GWAS phenotypes. In the second stage, a structural equation model is fitted to S by optimizing a diagonally weighted least squares (WLS) fit function that minimizes the difference between the model-implied genetic covariance matrix ∑(θ) and S, while considering the weights from V:


$$F_{WLS}(\theta )={\left(s-\sigma \;(\theta )\right)}^{\prime }D_{S}^{-1}(s-\sigma \;(\theta ))$$

where S and Σ(θ) are half-vectorized to form the vectors s and *σ*(*θ*), respectively. The matrix D_S_ is derived from the sampling covariance matrix of S, V_S_, by setting all off-diagonal elements to zero. The sampling covariance matrix of the stage 2 genomic SEM parameter estimates, V_θ_, is computed using a sandwich estimator:


$$V_{\theta }={{\left(\hat{\Delta },\Gamma^{-1}\hat{\Delta }\right)}^{\prime }}^{-1}\hat{\Delta },\Gamma^{-1}V_{S}\Gamma^{-1}\hat{\Delta }{\left(\hat{\Delta },\Gamma^{-1}\hat{\Delta }\right)}^{-1}$$

where Δ is the matrix of model derivatives evaluated at the parameter estimates, Γ is the stage 2 weight matrix, D_S_, and V_S_ is the sampling covariance matrix of S. Validation studies conducted by Grotzinger *et al.* (2019) demonstrated that genomic SEM produces unbiased standard errors, appropriately accounts for sample overlap in multivariate GWAS, and provides accurate point estimates for various population-generating models.

### 2.2 Overview of the Q_Trait_ function

**Figure 1.**
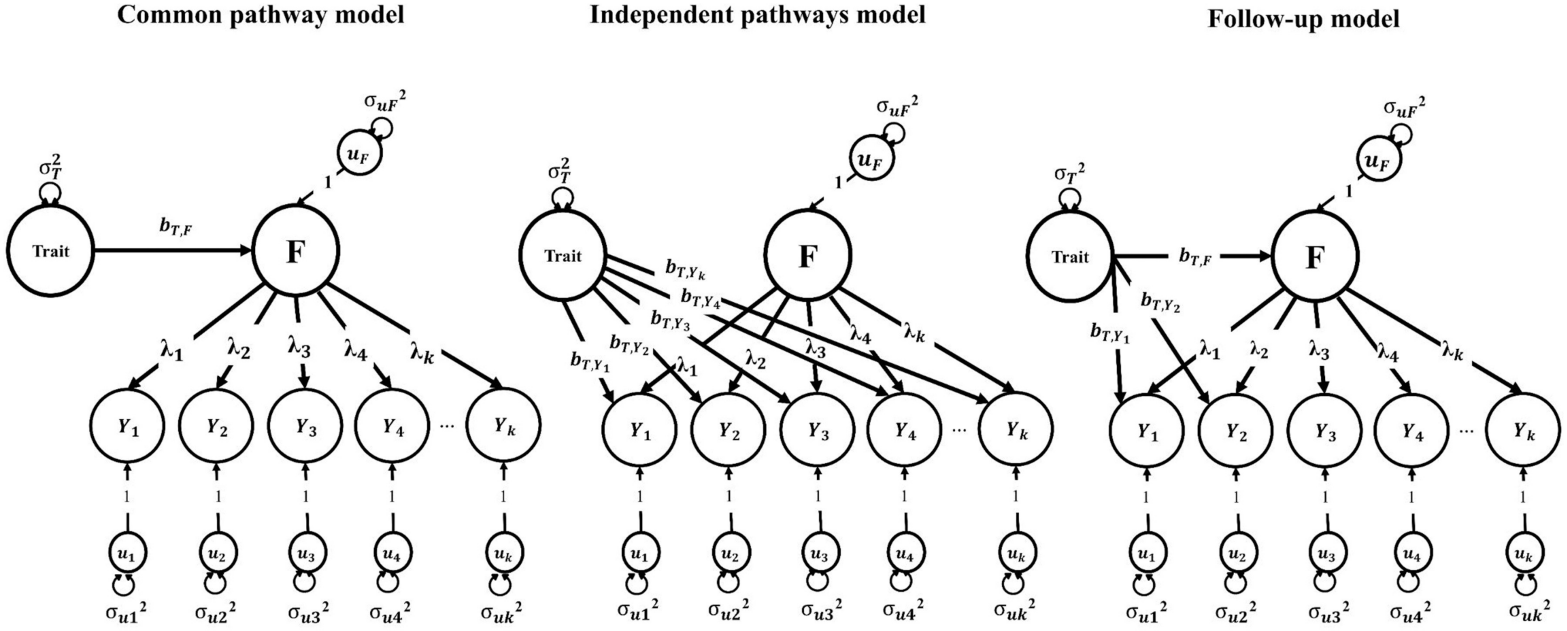
The fit of the common pathway and independent pathways models are compared for producing the Q_Trait_ heterogeneity index. The follow-up model is automatically re-specified by the Q_Trait_ function, allowing unconstrained direct paths to the identified outlying indicator traits (in this case Y_1_ and Y_2_). **F** represents the common factor underlying genetic covariances among the indicator traits. **Trait** represents the genetic component of the external GWAS correlate.

The Q_Trait_ function starts by fitting a genomic SEM common pathway model to estimate the regression coefficient relating the external correlate with the common factor (Fig. 1). The function determines the statistical significance of this parameter estimate using a Bonferroni-corrected *P*-value threshold based on the number of external correlates fed to the function. If the *P*-value of the regression weight of the external correlate on the common factor exceeds the Bonferroni-corrected threshold, the function then evaluates potential heterogeneity in the associations between the external correlate and the individual indicators loading on the common factor. To do so, the Q_Trait_ function incorporates two omnibus tests of heterogeneity: the Q_Trait_ statistic, which is based on statistical significance, and the local standardized root mean squared residual (lSRMR), an effect-size measure of heterogeneity. Both indices quantify heterogeneity in the associations between the external correlate of interest and the indicator phenotypes of the common factor. By integrating both statistical significance and effect size measures, the function avoids identifying negligible differences in high-powered comparisons and distinguishes meaningful differences from sampling variation in lower-powered comparisons.

### 2.3 Q_Trait_ statistic: an omnibus inferential test of heterogeneity

The Q_Trait_ statistic is based on the approach by Grotzinger *et al.* (2022) and evaluates whether a given factor adequately accounts for the patterns of associations between an external correlate and the individual traits loading on the factor. The Q_Trait_ statistic indexes the extent of model misfit for a common pathway model in which the effects of an external correlate on the individual phenotypes are specified to occur exclusively via a single effect of the external correlate on the latent factor, compared to a less restrictive independent pathways model where effects occur directly on each phenotype. A low Q_Trait_ indicates that the external correlate plausibly influences the latent factor, while a high Q_Trait_ suggests it does not.

Under the common pathway model, the expected effects of the external correlate on phenotype k (i.e. ${\hat{b}}_{\mathrm{Correlate},yk}$) can be expressed as $b_{\mathrm{Correlate},F}$ × $\lambda_{k}$, such that the effect of the correlate on each indicator is the product of its effect on the latent factor and the indicator’s factor loading:


$$\left[\begin{array}{c} {\hat{b}}_{\mathrm{Correlate},y1}\\ {\hat{b}}_{\mathrm{Correlate},y2}\\ {\hat{b}}_{\mathrm{Correlate},y3}\\ {\hat{b}}_{\mathrm{Correlate},y4}\\ \vdots \\ {\hat{b}}_{\mathrm{Correlate},yk} \end{array}]=b_{\mathrm{Correlate},F}[\begin{array}{c} \lambda_{1}\\ \lambda_{2}\\ \lambda_{3}\\ \lambda_{4}\\ \vdots \\ \lambda_{k} \end{array}\right]$$

where ${\hat{b}}_{\mathrm{Correlate},yk}$ corresponds with the expected effect of the external correlate on the indicator k, $b_{\mathrm{Correlate},F}$ is the effect of the external correlate on the latent factor F, and $\lambda_{k}$ denotes the loading of indicator k on the latent factor F. Misfit occurs when the vector of expected effects on phenotypes deviates from the vector of observed effects obtained from univariate GWAS. If the external correlate affects the individual phenotypes solely through the common factor, the vector of observed effects should be proportional to the vector of unstandardized loadings of those phenotypes, resulting in a low Q_Trait_. Conversely, if the observed effects are not proportional to the loadings, Q_Trait_ will be high, indicating model rejection.

When Q_Trait_ is high for a given external correlate, the linear association between the vector of univariate regression coefficients and the vector of unstandardized factor loadings will be weaker, potentially indicating one or more outlying indicators. It is important to note that we do not expect an external correlate acting exclusively on the common factor to have equal univariate associations with all phenotypes; this would only occur if the factor loadings were similar. Instead, if the external correlate truly acts exclusively on the common factor, we anticipate that the univariate associations will scale with the unstandardized factor loadings of the corresponding phenotypes. For example, a phenotype with a relatively low unstandardized factor loading should exhibit a correspondingly lower association with the external correlate compared to other phenotypes. If the association with that phenotype is comparable to associations with other phenotypes, this would contribute to a high Q_Trait_.

A χ^2^ difference test is used to compare the common and independent pathways models, as indicated by the Q_Trait_ heterogeneity index, which follows a χ^2^ distribution with K − 1 degrees of freedom (where K represents the number of indicator traits loading on the common factor). Q_Trait_ is computed as the difference in model fit between the independent pathways model and the common pathway model (where all associations are mediated by the factor):


$$Q_{\mathrm{Trait}}=\chi_{\mathrm{Independ}ent\;\mathrm{pathways}\;\mathrm{model}}^{2}-\;\chi_{\mathrm{Common}\;\mathrm{pathway}\;\mathrm{model}}^{2}$$

with degrees of freedom equal to (K − 1). A significant Q_Trait_ index suggests that the common factor does not adequately explain the pattern of associations between the individual traits and the external correlate. We note that we explore the regression coefficients of external correlates instead of Z statistics or *P*-values when investigating heterogeneity because differences in sample sizes, heritabilities, or polygenicity can lead to Z statistics that do not correlate with unstandardized factor loadings.

### 2.4 Local standardized root mean squared residual as an effect size measure of heterogeneity

To derive an effect size index of heterogeneity, we first define the residual genetic correlation (rg) for each indicator as the difference between the observed LDSC-derived genetic correlation and the genetic correlation implied by the common pathway model:


$${rg}_{\mathrm{Residual},k}=\;{rg}_{\mathrm{Observed},k}-{rg}_{\mathrm{Implied},k}$$

where ${rg}_{\mathrm{observed},k}$ is the LDSC-derived genetic correlation between the external correlate and indicator k, and ${rg}_{\mathrm{implied},k}$ is the corresponding model-implied genetic correlation under the common pathway. Positive or negative values of ${rg}_{\mathrm{residual},k}$ quantify the direction and magnitude by which indicator k departs from the association pattern predicted by the factor structure.

To summarize these deviations across all indicators, the local standardized root mean squared residual (lSRMR) is calculated as the root mean square of these residual genetic correlations (${rg}_{\mathrm{Residual},k})$:


$$\mathrm{lSRMR}=\sqrt{\frac{1}{K}\;\sum_{k=1}^{K}{\left({rG}_{\mathrm{Residual},k}\right)}^{2}}$$

where *K* is the number of indicator traits that load on the common factor, and ${rg}_{\mathrm{Residual},k}$ corresponds with the residual genetic correlation for indicator *k*. Larger lSRMR values indicate stronger average deviations from the common pathway model across indicators, whereas smaller values indicate closer alignment with the factor-predicted associations. The lSRMR is an adaptation of the local measure of parameter difference across groups (localSRMD) introduced by Schwaba *et al.* (2025) for comparing differences in genomic SEM parameters across subgroups of individuals (e.g. males and females). Unlike the original localSRMD, which focuses on parameter differences, the lSRMR computes the squared root mean residual correlation. Taken together, the residual correlation provides a local, indicator-specific diagnostic, while the lSRMR provides a global, standardized effect size of heterogeneity that is straightforward to report and compare across applications.

#### 2.4.1 Threshold selection and practical recommendations

To detect significant heterogeneity, our approach combines two complementary thresholds: (i) an absolute cutoff (default = 0.10 following SEM conventions; e.g. Hu and Bentler 1999, Bentler 2007), and (ii) a context-specific proportional cutoff (default = 25% of the average root mean squared genetic correlation), providing empirically calibrated sensitivity. To detect significant heterogeneity, the lSRMR must exceed both the absolute and context-specific thresholds. The absolute threshold reflects established benchmarks for identifying non-trivial misfit, helping to avoid trivial deviations especially in large GWAS samples. The context-specific threshold prioritizes practical significance by aligning with the principle of the “smallest effect size of interest” (SESOI; Lakens 2017, Anvari and Lakens 2021), ensuring deviations are meaningful within the empirical context.

We recommend starting with these default thresholds (0.10 absolute and 25% context-specific), but adjusting them according to study aims and researcher priorities. For instance, in some settings, identifying deviations from model expectations that are smaller than lSRMR=0.10, or less than 25% of the average root mean squared genetic correlation may be considered to be practically meaningful. The default thresholds are not universal rules but general recommendations. We provide sensitivity analyses applying alternative thresholds in our applications to Alzheimer’s disease and genetic *g*, illustrating their impact on outlying indicator detection (see Tables 1–4, available as supplementary data at *Bioinformatics* online).

**Table 1.** Heterogeneity statistics and patterns of associations between case-control GWAS of Alzheimer’s disease (AD) and proxy-phenotype GWAX of maternal and paternal history of AD in relation to 14 biobehavioral external correlates.

	Common pathway model			Follow-up model				
External correlate	rG (SE)	Q_Trait_ (df = 2)	lSRMR	rG (SE)	Q_Trait_ (df)	lSRMR	Reduction lSRMR (%)	Outlying indicators
Educational attainment	−0.08^a^ (0.03)	36.75^a^	0.13^b^	−0.16^a^	5.25 (1)	0.08^b^	33.85%	Maternal GWAX
Cognitive performance	−0.13^a^ (0.03)	26.61^a^	0.13^b^	−0.23^a^	1.57 (1)	0.06^b^	54.56%	Maternal GWAX
Ever smoker	−0.02 (0.03)	4.14	0.06^b^	–	–	–	–	–
Coronary artery disease	−0.15^a^ (0.04)	4.01	0.09^b^	–	–	–	–	–
LDL	0.02 (0.04)	3.69	0.03^b^	–	–	–	–	–
Lifespan	0.01 (0.04)	3.49	0.09^b^	–	–	–	–	–
HDL	0.11^a^ (0.04)	3.11	0.06^b^	–	–	–	–	–
Total brain volume	−0.04 (0.06)	2.24	0.08^b^	–	–	–	–	–
Loneliness	0.03 (0.05)	2.09	0.05^b^	–	–	–	–	–
Type 2 diabetes	−0.01 (0.03)	1.90	0.06^b^	–	–	–	–	–
White matter hyperintensities	0.04 (0.06)	0.53	0.05^b^	–	–	–	–	–
Heart failure	−0.19^a^ (0.06)	0.53	0.05	–	–	–	–	–
BMI	−0.05 (0.03)	0.23	0.01	–	–	–	–	–
Social deprivation	0.01 (0.07)	0.01	0.01^b^	–	–	–	–	–

a Statistically significant at the Bonferroni-corrected *P*-value threshold of *P* < 0.004.

b lSRMR surpassing 25% of the root mean square genetic correlation between the external correlate and the individual traits loading on the common factor.

### 2.5 Detection of outlying indicators and follow-up models

The Q_Trait_ function incorporates a method for identifying outlying indicators that deviate from the expectations under the common pathway model. These outlying indicators correspond to factor’s indicators for which the common factor does not adequately capture the association pattern with the external GWAS correlate.

Outlying indicator detection in the Q_Trait_ function relies on the same dual-threshold approach as the global effect-size-based heterogeneity assessment provided by the lSRMR. Specifically, indicator *k* is considered an outlying indicator if the residual genetic correlation, *rg*_Residual, k_, satisfies both of the following criteria:

1. The residual genetic correlation ${\mathrm{rg}}_{\mathrm{Residual},k}$ exceeds a predefined absolute threshold (default = 0.10).
2.
${\mathrm{rg}}_{\mathrm{Residual},k}$ also exceeds a context-specific threshold, set as 25% of the root mean square genetic correlation between the external correlate and all factor’s indicators:
  $${rg}_{\mathrm{Residu}al,k}>\mathrm{Absolute}\;\mathrm{threshold}\;$$
  and
  $${rg}_{\mathrm{Residual},k}>\mathrm{Context}-\mathrm{specific}\;\mathrm{threshold}\times \sqrt{\frac{1}{K}\;\sum_{k=1}^{K}{\left({rG}_{\mathrm{Correlate},k}\right)}^{2}}$$

This dual-threshold approach ensures that identified outlying indicators represent substantively meaningful deviations. By requiring that both criteria are met, trivial misfits are avoided and unnecessary model parameters are not introduced.

Detection of outlying indicators thus work in tandem, relying on both absolute and context-specific criteria. The absolute threshold helps prevent flagging minor deviations in high-powered datasets, while the context-specific threshold adapts detection sensitivity relative to the magnitude of genetic associations within the dataset. Researchers may wish to adjust these thresholds based on their specific trait characteristics and analytic goals; for example, lowering thresholds in low-signal settings to improve sensitivity, or raising them in high-signal settings to control false positives and preserve model parsimony. It may be advantageous to report sensitivity analyses using alternative thresholds to assess the robustness of detected outlying indicators.

When significant heterogeneity is detected, the Q_Trait_ function proceeds to fit a follow-up model that includes an unconstrained direct path from the external correlate to the most extreme outlying indicator. If the genetic correlation between the external correlate and the common factor remains significant in this follow-up model, and significant heterogeneity persists, the function selects the most extreme outlying indicator identified in that model and repeats the process until either the number of direct paths reaches saturation (i.e. K-1 direct paths are estimated, where K is the number of traits comprising the factor) or no significant heterogeneity is observed. If the number of unconstrained direct paths exceeds a substantial proportion of the total indicator traits (default = 50%), this suggests widespread heterogeneity, indicating that the common pathway model may no longer adequately represent the genetic associations. In such cases, it may be advisable to study the associations between the external correlate and individual indicator traits directly, rather than continuing to free additional paths in the model.

### 2.6 Sources of GWAS summary data for empirical analyses

#### 2.6.1 Cognitive function

We drew GWAS summary statistics obtained from the UK Biobank for seven cognitive tests: trail-making tests-B (*n* = 78 547), tower rearranging (*n* = 11 263), verbal numerical reasoning (VNR, *n* = 171 304), symbol digit substitution (*n* = 87 741), memory pairs-matching test (*n* = 331 679), matrix pattern recognition (*n* = 11 356), and reaction time (RT, *n* = 330 024). We employ the same common factor model described by de la Fuente *et al.* (2021), where a general dimension of shared genetic liability, termed genetic *g*, was identified in these same cognitive traits. Detailed information on the specific tests, participant criteria, and assessment details can be found in de la Fuente *et al.* (2021).

#### 2.6.2 Alzheimer’s disease

We compiled summary data from three published European ancestry direct case-control GWAS of AD and GWAX of parental history of AD. The direct case-control GWAS summary statistics included the discovery sample from the IGAP consortium (Kunkle *et al.* 2019), comprising 21 982 Clinical AD cases (mean age of onset = 72.93 years) and 41 944 controls (mean age of evaluation = 72.415 years). To address potential bias arising from variations in case prevalence across these cohorts, we adopted the approach outlined by Grotzinger *et al.* (2022), using the sum of effective sample sizes (4*v_k_*(1−*v_k_*))*n_k_*, where *v_k_* is the sample prevalence for each contributing GWAS to the meta-analysis. The GWAX summary data for proxy-phenotype AD included 27 696 cases and 260 980 controls for the maternal history of AD and 14 338 cases and 245 941 controls for the paternal history of AD, with both phenotypes sourced from the UK Biobank (Marioni *et al.* 2018). Case status in the UK Biobank was determined by responses to questions about parental Alzheimer’s disease/dementia history during the initial assessment visit (2006–2010), the first repeat assessment visit (2012–2013), and the imaging visit (2014+). Participants with parents younger than 60 years, those who died before reaching 60 years, and those lacking parental age information were excluded. Zhang *et al.* (2020) reported mean ages of 83.7 years for maternal cases, 81.8 years for paternal cases, 78.1 years for maternal controls, and 76.2 years for paternal controls. Further details on case ascertainment, genotyping, and quality control can be found in the original articles providing the corresponding summary statistics.

#### 2.6.3 External correlates

To explore the generality versus specificity of associations between the general intelligence and the Alzheimer’s disease factors, we selected external GWAS correlates based on their relevance for cognitive function, health related lifestyle and general liability for disease. The external correlates included educational attainment (*n* = 3 037 499; Okbay *et al.* 2022), cognitive performance (*n* = 257 841; Lee *et al.* 2018), total brain volume (*n* = 36 778; Fürtjes *et al.* 2023), white matter hyperintensities (*n* = 50 970; Sargurupremraj *et al.* 2020), ever smoker (*n* = 518 633; Liu *et al.* 2019), loneliness (*n* = 452 302; Day *et al.* 2018), social deprivation (*n* = 112 151; Hill *et al.* 2016), parental lifespan (*n* = 1 012 240; Timmers *et al.* 2019), BMI (*n* = 681 275; Yengo *et al.* 2018), HDL (*n* = 1 320 016; Graham *et al.* 2021), LDL (*n* = 1 320 016; Graham *et al.* 2021), coronary artery disease (*n* = 1 378 170; van der Harst and Verweij 2018), heart failure (*n* = 933 970; Shah *et al.* 2020), and Type 2 diabetes (*n* = 933 970; Mahajan *et al.* 2022).

For the specific analysis of the genetic *g* factor, we included neuropsychiatric and personality traits given their relevance for cognitive function reported in the literature. Neuropsychiatric disorders data were obtained mostly from the psychiatric genetics consortium (PGC) database (Sullivan 2010), including schizophrenia (Neff = 117 494; Trubetskoy *et al.* 2022), bipolar disorder (Neff = 101 963; Mullins *et al.* 2021), autism spectrum disorder (Neff = 43 778; Grove *et al.* 2019), attention-deficit hyperactivity disorder (Neff = 103 135; Demontis *et al.* 2023). Personality traits were also considered. We used GWAS summary statistics for openness to experience (n = 220 015) and conscientiousness (*n* = 234 880) from Gupta *et al.* (2024). These data represent a meta-analysis of the Million Veteran Program (MVP) release version 4 together with previous GWAS of the Big Five in other cohorts. In the MVP, traits were measured using the BFI-10, a 10-item self-report personality scale administered as part of the Lifestyle survey to participants of primarily European ancestry (mean age ≈ 65.5 years, 8% female).

## 3 Results

### 3.1 Alzheimer’s disease

In the analysis of Alzheimer’s disease, 14 external correlates were considered, out of which five exhibited significant associations with the AD factor at the Bonferroni-corrected *P*-value threshold of *P* < 0.004 (Table 1). These include educational attainment (EA), cognitive performance, high-density lipoprotein (HDL), coronary artery disease (CAD), and heart failure. Among these, HDL (rG = 0.11, SE = 0.04, *P*=0.002), CAD (rG = −0.15, SE = 0.04, *P* = 0.001), and heart failure (rG = −0.19, SE = 0.06, *P* = 0.001) did not exhibit significant deviations from the common pathway model according to the omnibus tests of heterogeneity, suggesting that these external correlates are broadly relevant for both the direct GWAS and the family GWAX AD phenotypes (Table 1). On the contrary, we found evidence of heterogeneity in the associations between EA (Q_Trait_ (2) = 36.75, *P* < 0.001; lSRMR = 0.13), cognitive performance (Q_Trait_ (2) = 26.61, *P* < 0.001; lSRMR = 0.11) and the individual AD phenotypes, indicating that these external correlates may operate in more specific pathways with respect to direct GWAS and proxy GWAX of AD.

**Figure 2.**
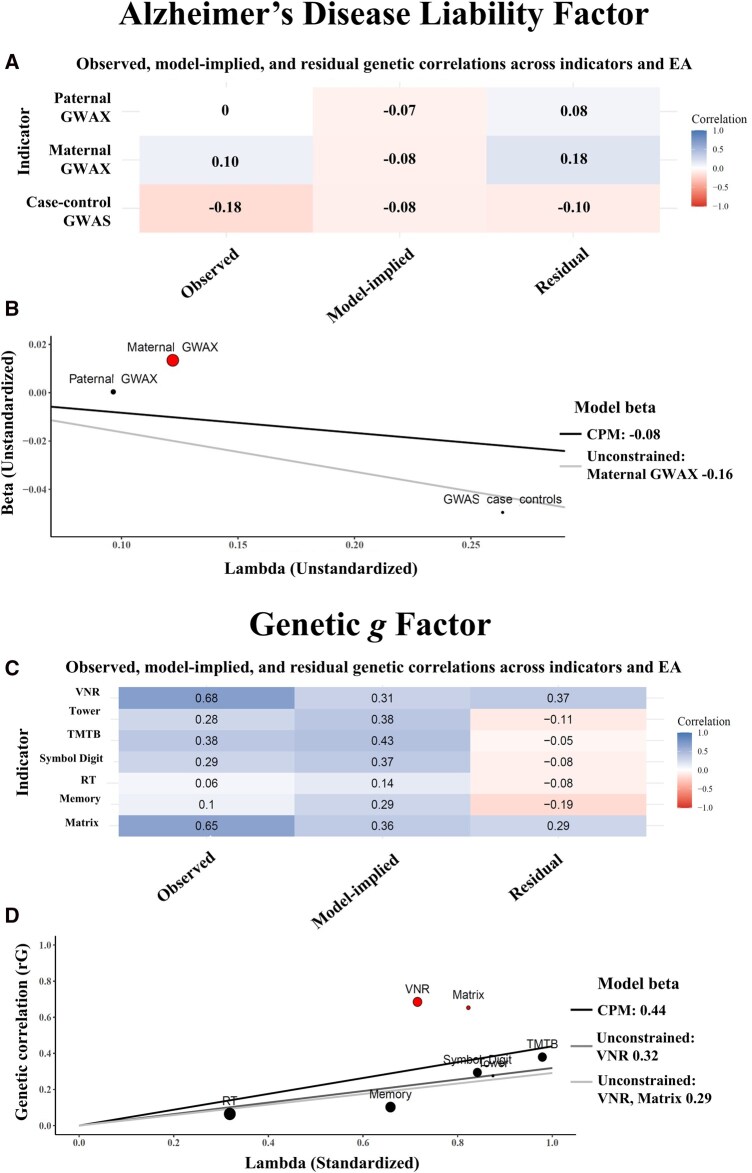
(A) Observed, model-implied, and residual genetic correlations between the three AD indicators and educational attainment (EA). (B) Scatterplot of unstandardized betas of EA on AD indicators against their unstandardized loadings on the AD factor (de la Fuente *et al.* 2021); dot size reflects inverse variance. (C) Observed, model-implied, and residual genetic correlations between seven cognitive indicators and EA. (D) Scatterplot of genetic correlations between EA and cognitive indicators against their standardized loadings on the genetic g factor; dot size reflects inverse variance. CPM, common pathway model.

The Q_Trait_ statistic tests the null hypothesis that the genetic associations between each external correlate (e.g. EA, cognitive performance) and the individual AD indicators are fully accounted for by the common pathway model. A non-significant Q_Trait_ indicates that the common pathway adequately explains these associations for a given trait. A significant Q_Trait_ indicates that the common factor does not fully capture the genetic association patterns between the trait and the individual GWAS and GWAX AD indicators, suggesting additional trait-specific genetic effects beyond the common factor. The path diagrams of the common pathway models for EA and the AD factor are shown in Figs 1 and 2, available as supplementary data at *Bioinformatics* online.

The outlying indicator detection method indicated that maternal GWAX presented significant deviations from the common pathway model (Fig. 3, available as supplementary data at *Bioinformatics* online). Specifically, maternal GWAX of AD presented positive associations with both EA and cognitive performance (implying that genetic propensities toward higher educational attainment and higher cognitive function are associated with greater risk of AD) whereas the common pathway model implied that these associations would be negative (genetic propensities toward higher EA and higher cognitive function are protective against AD). The follow-up models for educational attainment (Q_Trait_ (1) = 5.25, *P* = 0.022; lSRMR = 0.08) and cognitive performance (Q_Trait_ (1) = 1.57, *P* = 0.260; lSRMR = 0.06) with unconstrained direct paths to maternal AD GWAX did not reveal significant residual heterogeneity, while the genetic associations of EA and cognitive performance with lower risk of AD remained statistically significant for both educational attainment (rG = −0.16, SE = 0.03, *P* = 0.001) and cognitive performance (rG = −0.23, SE = 0.04, *P* < 0.001).

Figure 2 contains plots presenting (A) the observed, model-implied (common pathway model), and residual genetic correlations between EA and the three indicators of the AD liability factor—maternal GWAX, paternal GWAX, and case-control GWAS of AD, and (B) a scatterplot of unstandardized beta coefficients of EA on the three indicators against their respective unstandardized factor loadings on the AD liability factor (de la Fuente *et al.* 2022). The size of each dot corresponds to the inverse of the variance of the unstandardized beta coefficient. The figure also shows the model-implied association between educational attainment and AD liability from both the common pathway and the unconstrained model with direct path to maternal GWAX, represented by the slope of the regression line.

The common pathway model estimates the genetic association between EA and AD liability but fails to fully account for the relations with maternal GWAX indicator, as indicated in Fig. 2 by the deviation of the maternal GWAX from the line representing the model-implied association from the common pathway model, and the large residual genetic correlation between maternal GWAX and EA (0.18). If the common pathway model were sufficient, genetic correlations with EA would scale proportionally with the factor loadings of the indicators on the AD liability factor. However, maternal GWAX deviates from this expectation, indicating stronger-than-expected positive genetic correlation with EA. This suggests that the association between EA and maternal GWAX involves additional genetic effects beyond those captured by the common pathway model. These findings highlight the limitations of the common pathway and imply that the genetic relationships between EA and AD differs depending on whether direct case-control GWAS versus proxy GWAX of parental history are used. It is possible that the maternal GWAX in particular suffers from a pernicious form of ascertainment bias. It can be seen from Fig. 2 that when the model is relaxed to account for the specific genetic relation between EA and the maternal GWAX, the model-implied genetic association between EA and AD doubles in strength.

### 3.2 Cognitive function

We further investigated the generality versus specificity of associations between 20 external GWAS correlates (the same 14 traits analyzed in AD, and 6 additional neuropsychiatric phenotypes associated with cognitive function) and the genetic general intelligence factor (*g*) described in de la Fuente *et al.* (2021). The genetic *g* factor accounts for the shared genetic variance among seven continuous cognitive trait GWAS phenotypes. Out of the 20 external correlates considered, 15 exhibited significant associations with the genetic *g* factor at the Bonferroni-corrected *P*-value threshold of *P* < 0.002 (Table 2). The five external correlates that exhibited significant heterogeneity were: educational attainment (EA), cognitive performance, loneliness, lifespan, and attention-deficit/hyperactivity disorder (ADHD), suggesting more specific pathways with respect to genetic *g*. Notably, several other external correlates—including bipolar disorder, total brain volume, ever smoker, loneliness, BMI, HDL, LDL, CAD, heart failure, and T2DM—showed statistically significant Q_Trait_ values but had lSRMR measures below the 25% threshold. When applying the combined Q_Trait_ and lSRMR dual-threshold criterion, these cases were not considered to represent meaningful heterogeneity that would justify freeing additional direct genetic paths.

**Table 2.** Heterogeneity statistics and patterns of associations between the g factor in relation to 20 biobehavioral external correlates.

	Common pathway model			Follow-up model				
External correlate	rG (SE)	Q_trait_ (df = 6)	lSRMR	rG (SE)	Q_Trait_ (df)	lSRMR	Reduction lSRMR (%)	Outlying indicators
Cognitive performance	0.82^a^ (0.03)	2892.15^a^	0.18^b^	0.72^a^ (0.03)	49.18^a^ (5)	0.13	29.37%	VNR
Educational attainment	0.44^a^ (0.02)	1424.50^a^	0.20^b^	0.29^a^ (0.02)	40.23^a^ (4)	0.06	70.92%	VNR, Matrix
ADHD	−0.28^a^ (0.03)	202.12^a^	0.13^b^	−0.14^a^ (0.03)	31.47^a^ (4)	0.07	50.10%	VNR, Matrix
BMI	−0.07^a^ (0.02)	101.61^a^	0.10^b^	–	–	–	–	–
Ever smoker	−0.13^a^ (0.02)	73.22^a^	0.09	–	–	–	–	–
Openness to experience	0.02 (0.04)	70.55^a^	0.12^b^	–	–	–	–	–
Social deprivation	−0.23^a^ (0.05)	64.72^a^	0.12^b^	−0.22^a^ (0.05)	69.12^a^ (5)	0.10	19.44%	Matrix
Autism	0.08 (0.04)	62.21^a^	0.10^b^	–	–	–	–	–
Type 2 diabetes	−0.07^a^ (0.02)	58.63^a^	0.07	–	–	–	–	–
Lifespan	0.25^a^ (0.03)	51.93^a^	0.11^b^	0.24^a^ (0.03)	41.75^a^ (5)	0.07	36.87%	Matrix
Loneliness	−0.15^a^ (0.03)	43.83^a^	0.07	–	–	–	–	–
Coronary artery disease	−0.11^a^ (0.02)	42.95^a^	0.07	–	–	–	–	–
Bipolar disorder	−0.25^a^ (0.03)	32.26^a^	0.07	–	–	–	–	–
Heart failure	−0.16^a^ (0.04)	27.01^a^	0.09	–	–	–	–	–
LDL	0.00 (0.03)	23.82^a^	0.06	–	–	–	–	–
HDL	0.04 (0.03)	23.73^a^	0.06	–	–	–	–	–
Total brain volume	0.21^a^ (0.04)	20.82^a^	0.07	–	–	–	–	–
Schizophrenia	−0.39^a^ (0.03)	17.40	0.06	–	–	–	–	–
Conscientiousness	−0.12^a^ (0.04)	14.48	0.08	–	–	–	–	–
White matter hyperintensities	−0.11 (0.04)	9.02	0.05	–	–	–	–	–

a Statistically significant at the Bonferroni-corrected *P*-value threshold of *P* < 0.002.

b lSRMR surpassing 25% of the root mean square genetic correlation between the external correlate and the individual traits loading on the common factor.

We focus our attention on the three external traits presenting the highest genetic correlations with the g factor: educational attainment (rG = 0.44, *P* < 0.001), cognitive performance (rG = 0.82, *P* < 0.001), and ADHD (rG = −0.28, *P* < 0.001). The path diagram of the common pathway model for EA and the genetic *g* factor is shown in Fig. 2, available as supplementary data at *Bioinformatics* online. According to outlying indicator detection method, VNR and Matrix showed stronger positive genetic associations with EA and cognitive performance than those expected from the common pathway model. In contrast, the genetic correlations between these cognitive traits and ADHD were significantly more negative than those implied by the common pathway model. Follow-up models for ADHD [Q_Trait_ (4) = 31.47, *P* < 0.001; lSRMR = 0.07] and EA [Q_Trait_ (4) = 40.23, *P* < 0.001; lSRMR = 0.06] with unconstrained direct paths to VNR, Matrix, and for cognitive performance [cognitive performance (Q_Trait_ (5) = 49.18, *P* < 0.001; lSRMR = 0.13] with unconstrained direct path to VNR, did not reveal significant heterogeneity, while the associations with the *g* factor were weaker than those estimated under the common pathway model, albeit remaining statistically significant (Table 2).

The common pathway model estimates the genetic association between EA and general cognitive function (genetic *g*) but fails to fully capture the pattern of associations across all cognitive indicators. As shown in Fig. 2, both the VNR and Matrix reasoning tests deviate notably from the line representing the model-implied association, exhibiting substantial residual genetic correlations with EA (0.37 and 0.29, respectively). If the common pathway model adequately accounted for the genetic correlations between EA and these cognitive traits, the observed correlations would scale proportionally with the indicators’ factor loadings on genetic *g*. However, VNR and Matrix reasoning display stronger-than-expected associations, suggesting the presence of additional trait-specific genetic effects. When the model is relaxed to allow for direct genetic associations between EA and these two indicators, the model-implied association between EA and the general cognitive factor is attenuated, decreasing from 0.44 to 0.29. These findings suggest that a more nuanced or multifactorial representation of genetic effects on cognitive traits may be warranted.

## 4 Discussion

This paper extends the genomic SEM framework by extending the Q_Trait_ method for investigating heterogeneity of genetic associations between external correlates and the indicator traits of a common factor. By incorporating both global statistical significance and effect size measures, our approach overcomes the limitations of sole reliance on statistical significance, which can either obscure meaningful differences in low-power studies or detect trivial differences in high-power studies. Furthermore, our method enables the identification of specific outlying indicator GWAS phenotypes that deviate from the expectations of the common pathway model, revealing genetic pathways that may not be captured by effects exclusively on the factor. This is particularly critical, as such heterogeneity could pose a threat to the external validity of genetic factor models—an issue that has often been underappreciated in the literature. By providing a more refined approach to assess how well these models approximate the genetic relationships between complex traits and external correlates, our method offers a more comprehensive and robust framework for understanding the genetic architecture of complex traits.

Combining direct case-control GWAS with proxy-phenotype GWAS (GWAX), such as maternal and paternal AD history, is a widely used strategy to boost statistical power in AD research (Bellenguez *et al*. 2022, Hujoel *et al.* 2020, Liu 2022). However, genetic associations between AD and external biobehavioral traits, such as educational attainment (EA) and cognitive performance, can differ between direct case-control GWAS and proxy GWAX. Wu *et al.* (2024) employed GWAS-by-subtraction (Demange *et al*. 2021) to disentangle these differences, a method particularly suited for comparing pairs of traits. In contrast, our approach extends beyond pairwise comparisons, allowing for the integration of multiple indicators while automating the identification of misfit sources. Additionally, it provides a framework for testing the validity of inference within the factor model—an aspect not addressed by GWAS-by-subtraction. We observed significant and substantial heterogeneity in the genetic associations between both EA and cognitive performance, and AD. Maternal AD history displayed anomalous positive genetic correlations with both EA and cognitive performance, despite negative genetic correlations between case-control AD, cognitive performance, and EA. Our results indicate that an analysis naïve to this heterogeneity underestimated the associations between both educational attainment and cognitive performance and lower AD risk. However, this bias was automatically detected and corrected for using the function introduced here. Several hypotheses may explain divergent results with respect to direct and proxy GWAS data (de la Fuente *et al.* 2021, Escott-Price and Hardy 2022). First, proxy GWAX data could be influenced by biases in family health history reporting, such as individuals misremembering or confusing their parents’ disease status, potentially attenuating or contaminating the heritability of the proxy phenotype with other heritable traits. Second, differences in diagnostic quality and criteria between proxy reports of historical disease status and the carefully screened case-control samples used in direct GWAS could also bias the proxy data. Finally, proxy GWAX may capture additional genetic signals not directly related to AD, including the pernicious effects of sample bias, which may itself be differential according to unmeasured heritable phenotypes (Pirastu *et al.* 2021, Schoeler *et al.* 2023). These findings and hypotheses underscore the need for careful interpretation of proxy GWAS data, distinguishing genetic influences specific to AD from those associated with other familial traits. Recent advances such as synthetic surrogate (SynSurr) analysis provide a framework for valid inference by jointly modeling observed and surrogate phenotypes, and could be extended to liability-scale models for proxy-based AD studies, potentially reducing heterogeneity inherent in GWAX summary statistics (McCaw *et al.* 2024).

Our analysis of the genetic *g* factor revealed significant heterogeneity in its associations with various external correlates, shedding light on the complex genetic underpinnings of general cognitive ability. Among the 20 external correlates examined, 15 showed significant associations with the genetic g factor, and 5 of these exhibited heterogeneity, suggesting more specific genetic pathways. These included EA, cognitive performance, loneliness, lifespan, and attention-deficit/hyperactivity disorder (ADHD). This heterogeneity indicates that while these traits are linked to cognitive ability, their genetic influences likely vary across different cognitive domains. For example, verbal numerical reasoning (VNR) showed stronger genetic associations with educational attainment and cognitive performance than expected by the common pathway model. This may be due to the significant crystallized knowledge component of the VNR test (Fawns-Ritchie and Deary 2020, de la Fuente *et al.* 2021, Londoño-Correa *et al.* 2025), which aligns more closely with educational measures like math ability and degree level. Importantly, these association patterns were further clarified through outlying indicator detection and correction. Specifically, the association between educational attainment and the genetic *g* factor was smaller in the corrected model, in contrast to the AD case, where correction led to a stronger association. This highlights that outlying indicator detection does not systematically inflate or deflate associations, but rather corrects for bias in either direction.

While our method provides valuable insights, several limitations should be considered. First, the use of GWAS summary statistics means that our results are dependent on the quality of the underlying data, which can be influenced by factors such as sample size and bias, genotyping and phenotyping errors, and population stratification (Wainschtein *et al.* 2022). These factors can introduce biases, especially in studies with smaller samples or less accurate genetic data (Dastani *et al.* 2017). Another consideration is assortative mating, where genetic similarities between partners can inflate heritability estimates and alter patterns of genetic associations across traits, potentially distorting the observed relationships between traits (Barban *et al.* 2016, Border *et al.* 2022). These limitations highlight the importance of interpreting genetic correlations with caution and recognizing the influence of biases that may shape these associations.

In conclusion, our study introduces a novel and efficient framework for assessing the external validity of genetic factor models by evaluating how well they approximate the genetic relationships across constellations of external complex traits. By incorporating both statistical significance and effect size measures, our method provides a more comprehensive understanding of genetic correlations, overcoming the limitations of traditional approaches that rely solely on statistical significance. This framework’s ability to identify genetic heterogeneity and detect outlying indicator phenotypes not only offers valuable insights into the genetic architecture of complex traits but also facilitates the generation of mechanistic hypotheses about genetic associations. By highlighting areas where genetic factor models may not fully capture the underlying genetic relationships, the identification of outlying indicators can inform targeted investigations into specific genetic mechanisms.

## Supplementary Material

btaf568_Supplementary_Data
