# Supplementary material for: Distinguishing specific from broad genetic associations between external correlates and common factors
Source: Bioinformatics. 2025 Oct 14;41(11):btaf568. doi: 10.1093/bioinformatics/btaf568 (PMC12597881; doi:10.1093/bioinformatics/btaf568)
Supplement: btaf568_Supplementary_Data [file btaf568_supplementary_data.docx]

**Supplementary Materials for:**

**Distinguishing Specific from Broad Genetic Associations between External Correlates and Common Factors**

Javier de la Fuente^1*^, Diego Londoño-Correa^1^ and Elliot M. Tucker-Drob^1^

^1^Department of Psychology, The University of Texas at Austin, Austin, TX, USA

*Corresponding author: Department of Psychology, University of Texas at Austin, 108 E Dean Keeton St, Austin, TX 78712, EE. UU. E-mail: [j.delafuente@utexas.edu](mailto:j.delafuente@utexas.edu)

**Table S1.** Heterogeneity statistics and patterns of associations between case-control GWAS of Alzheimer’s disease (AD) and proxy-phenotype GWAX of maternal and paternal history of AD in relation to 14 biobehavioral external correlates using liberal thresholds for heterogeneity and outlying indicator detection (i.e., absolute threshold = .08; context-specific threshold = .15).

|  | **Common pathway model** | | | **Follow-up model** | | |  |  |
| --- | --- | --- | --- | --- | --- | --- | --- | --- |
| **External correlate** | **rG (SE)** | **QTrait (df)** | **lSRMR** | **rG (SE)** | **QTrait (df)** | **lSRMR** | **Reduction lSRMR (%)** | **Outlying indicators** |
| Educational attainment | -0.08* (0.03) | 36.75* (2) | 0.13a | -0.16* | 5.25 (1) | 0.08a |  | Maternal GWAX |
| Cognitive performance | -0.13* (0.03) | 26.61* (2) | 0.13a | -0.23* | 1.57 (1) | 0.06 |  | Maternal GWAX |
| Total brain volume | -0.04 (0.06) | 2.24 (2) | 0.08a | - | - | - |  | - |
| White matter hyperintensities | 0.04 (0.06) | 0.53 (2) | 0.05 | - | - | - |  | - |
| Ever smoker | -0.02 (0.03) | 4.14 (2) | 0.06 | - | - | - |  | - |
| Loneliness | 0.03 (0.05) | 2.09 (2) | 0.05 | - | - | - |  | - |
| Social deprivation | 0.01 (0.07) | 0.01 (2) | 0.01 | - | - | - |  | - |
| Lifespan | 0.01 (0.04) | 3.49 (2) | 0.09a | - | - | - |  | - |
| BMI | -0.05 (0.03) | 0.23 (2) | 0.01 | - | - | - |  | - |
| HDL | 0.11* (0.04) | 3.11 (2) | 0.06 | - | - | - |  | - |
| LDL | 0.02 (0.04) | 3.69 (2) | 0.03 | - | - | - |  | - |
| Coronary artery disease | -0.15* (0.04) | 4.01 (2) | 0.09a | - | - | - |  | - |
| Heart failure | -0.19* (0.06) | 0.53 (2) | 0.05 | - | - | - |  | - |
| Type 2 diabetes | -0.01 (0.03) | 1.90 (2) | 0.06 | - | - | - |  | - |

*^*^Statistically significant at the Bonferroni-corrected p-value threshold of p < .004.
^a^lSRMR surpassing 15% of the root mean square genetic correlation between the external correlate and the individual traits loading on the common* factor.

**Table S2.** Heterogeneity statistics and patterns of associations between the g factor in relation to 20 biobehavioral external correlates using conservative thresholds for heterogeneity and outlying indicator detection (i.e., absolute threshold = .15; context-specific threshold = .35).

|  | **Common pathway model** | | | **Follow-up model** | | |  |  |
| --- | --- | --- | --- | --- | --- | --- | --- | --- |
| **External correlate** | **rG (SE)** | **QTrait (df)** | **lSRMR** | **rG (SE)** | **QTrait (df)** | **lSRMR** | **Reduction lSRMR (%)** | **Outlying indicators** |
| Educational attainment | -0.08* (0.03) | 36.75* (2) | 0.13 | - | - | - |  | - |
| Cognitive performance | -0.13* (0.03) | 26.61* (2) | 0.13 | - | - | - |  | - |
| Total brain volume | -0.04 (0.06) | 2.24 (2) | 0.08 | - | - | - |  | - |
| White matter hyperintensities | 0.04 (0.06) | 0.53 (2) | 0.05 | - | - | - |  | - |
| Ever smoker | -0.02 (0.03) | 4.14 (2) | 0.06 | - | - | - |  | - |
| Loneliness | 0.03 (0.05) | 2.09 (2) | 0.05 | - | - | - |  | - |
| Social deprivation | 0.01 (0.07) | 0.01 (2) | 0.01 | - | - | - |  | - |
| Lifespan | 0.01 (0.04) | 3.49 (2) | 0.09 | - | - | - |  | - |
| BMI | -0.05 (0.03) | 0.23 (2) | 0.01 | - | - | - |  | - |
| HDL | 0.11* (0.04) | 3.11 (2) | 0.06 | - | - | - |  | - |
| LDL | 0.02 (0.04) | 3.69 (2) | 0.03 | - | - | - |  | - |
| Coronary artery disease | -0.15* (0.04) | 4.01 (2) | 0.09 | - | - | - |  | - |
| Heart failure | -0.19* (0.06) | 0.53 (2) | 0.05 | - | - | - |  | - |
| Type 2 diabetes | -0.01 (0.03) | 1.90 (2) | 0.06 | - | - | - |  | - |

rG = genetic correlation. lSRMR = local standardized mean squared residual.
*Statistically significant at the Bonferroni-corrected p-value threshold of p < .002.
^a^lSRMR surpassing 35% of the root mean square genetic correlation between the external correlate and the individual traits loading on the common factor

**Table S3.** Heterogeneity statistics and patterns of associations between the g factor in relation to 20 biobehavioral external correlates using liberal thresholds for heterogeneity and outlying indicator detection (i.e., absolute threshold = .08; context-specific threshold = .15).

|  | **Common pathway model** | | | **Follow-up model** | | |  |  |
| --- | --- | --- | --- | --- | --- | --- | --- | --- |
|  | **rG (SE)** | **QTrait statistic (df)** | **lSRMR** | **rG (SE)** | **QTrait statistic (df)** | **lSRMR** | **Reduction lSRMR (%)** | **Outying indicators** |
| Schizophrenia | -0.39* (0.03) | 17.40 (6) | 0.06 | - | - | - | - | - |
| Bipolar disorder | -0.25* (0.03) | 32.26* (6) | 0.07 | - | - | - | - | - |
| Autism | 0.08 (0.04) | 62.21* (6) | 0.10a | - | - | - | - | - |
| ADHD | -0.28* (0.03) | 202.12* (6) | 0.13a | -0.14* (0.03) | 31.47 (4) | 0.07 | 50.10% | VNR,Matrix |
| Openess to experience | 0.02 (0.04) | 70.55* (6) | 0.12a | - | - | - | - | - |
| Conscientiousness | -0.12* (0.04) | 14.48 (6) | 0.08 | - | - | - | - | - |
| Educational attainment | 0.44* (0.02) | 1424.50* (6) | 0.20a | 0.29* (0.02) | 40.23 (4) | 0.06 | 70.12% | VNR,Matrix |
| Cognitive performance | 0.82* (0.03) | 2892.15* (6) | 0.18a | 0.70* (0.02) | -0.38 (4) | 0.09 | 51.12% | VNR,Matrix |
| Total brain volume | 0.21* (0.04) | 20.82* (6) | 0.07 | - | - | - | - | - |
| White matter hyperintensities | -0.11 (0.04) | 9.02 (6) | 0.05 | - | - | - | - | - |
| Ever smoker | -0.13* (0.02) | 73.22* (6) | 0.09a | -0.12* (0.02) | 77.32 (5) | 0.06 | 31.80% | Matrix |
| Loneliness | -0.15* (0.03) | 43.83* (6) | 0.07 | - | - | - | - | - |
| Social deprivation | -0.23* (0.05) | 64.72* (6) | 0.12a | -0.11 (0.05) | 18.23 (4) | 0.08a | 31.54% | Matrix,VNR |
| Lifespan | 0.25* (0.03) | 51.93* (6) | 0.11a | 0.24* (0.03) | 41.75 (5) | 0.07 | 36.87% | Matrix |
| BMI | -0.0*7 (0.02) | 101.61* (6) | 0.10a | -0.11* (0.02) | 56.27 (5) | 0.07 | 30.08% | Memory |
| HDL | 0.04 (0.03) | 23.73* (6) | 0.06 | - | - | - | - | - |
| LDL | 0.00 (0.03) | 23.82* (6) | 0.06 | - | - | - | - | - |
| Coronary artery disease | -0.11* (0.02) | 42.95* (6) | 0.07 | - | - | - | - | - |
| Heart failure | -0.16* (0.04) | 27.01* (6) | 0.09a | -0.20* (0.04) | 10.77 (5) | 0.07 | 24.12% | Memory |
| Type 2 diabetes | -0.07* (0.02) | 58.63* (6) | 0.07 | - | - | - | - | - |

rG = genetic correlation. lSRMR = local standardized mean squared residual.
*Statistically significant at the Bonferroni-corrected p-value threshold of p < .002.
^a^lSRMR surpassing 15% of the root mean square genetic correlation between the external correlate and the individual traits loading on the common factor

**Table S4.** Heterogeneity statistics and patterns of associations between the g factor in relation to 20 biobehavioral external correlates using conservative thresholds for heterogeneity and outlying indicator detection (i.e., absolute threshold = .15; context-specific threshold = .35).

|  | **Common pathway model** | | | **Follow-up model** | | |  |  |
| --- | --- | --- | --- | --- | --- | --- | --- | --- |
|  | **rG (SE)** | **QTrait statistic (df)** | **lSRMR** | **rG (SE)** | **QTrait statistic (df)** | **lSRMR** | **Reduction lSRMR (%)** | **Outying indicators** |
| Schizophrenia | -0.39* (0.03) | 17.40 (6) | 0.06 | - | - | - | - | - |
| Bipolar disorder | -0.25* (0.03) | 32.26* (6) | 0.07 | - | - | - | - | - |
| Autism | 0.08 (0.04) | 62.21* (6) | 0.10 | - | - | - | - | - |
| ADHD | -0.28* (0.03) | 202.12* (6) | 0.13 | - | - | - | - | - |
| Openess to experience | 0.02 (0.04) | 70.55* (6) | 0.12 | - | - | - | - | - |
| Conscientiousness | -0.12* (0.04) | 14.48 (6) | 0.08 | - | - | - | - | - |
| Educational attainment | 0.44* (0.02) | 1424.50* (6) | 0.20^a^ | 0.29 (0.02) | 40.23 (4) | 0.06 | 70.92% | VNR,Matrix |
| Cognitive performance | 0.82* (0.03) | 2892.15* (6) | 0.18 | - | - | - | - | - |
| Total brain volume | 0.21* (0.04) | 20.82* (6) | 0.07 | - | - | - | - | - |
| White matter hyperintensities | -0.11 (0.04) | 9.02 (6) | 0.05 | - | - | - | - | - |
| Ever smoker | -0.13* (0.02) | 73.22* (6) | 0.09 | - | - | - | - | - |
| Loneliness | -0.15* (0.03) | 43.83* (6) | 0.07 | - | - | - | - | - |
| Social deprivation | -0.23* (0.05) | 64.72* (6) | 0.12 | - | - | - | - | - |
| Lifespan | 0.25* (0.03) | 51.93* (6) | 0.11 | - | - | - | - | - |
| BMI | -0.0*7 (0.02) | 101.61* (6) | 0.10 | - | - | - | - | - |
| HDL | 0.04 (0.03) | 23.73* (6) | 0.06 | - | - | - | - | - |
| LDL | 0.00 (0.03) | 23.82* (6) | 0.06 | - | - | - | - | - |
| Coronary artery disease | -0.11* (0.02) | 42.95* (6) | 0.07 | - | - | - | - | - |
| Heart failure | -0.16* (0.04) | 27.01* (6) | 0.09 | - | - | - | - | - |
| Type 2 diabetes | -0.07* (0.02) | 58.63* (6) | 0.07 | - | - | - | - | - |

rG = genetic correlation. lSRMR = local standardized mean squared residual.
*Statistically significant at the Bonferroni-corrected p-value threshold of p < .002.
^a^lSRMR surpassing 35% of the root mean square genetic correlation between the external correlate and the individual traits loading on the common factor


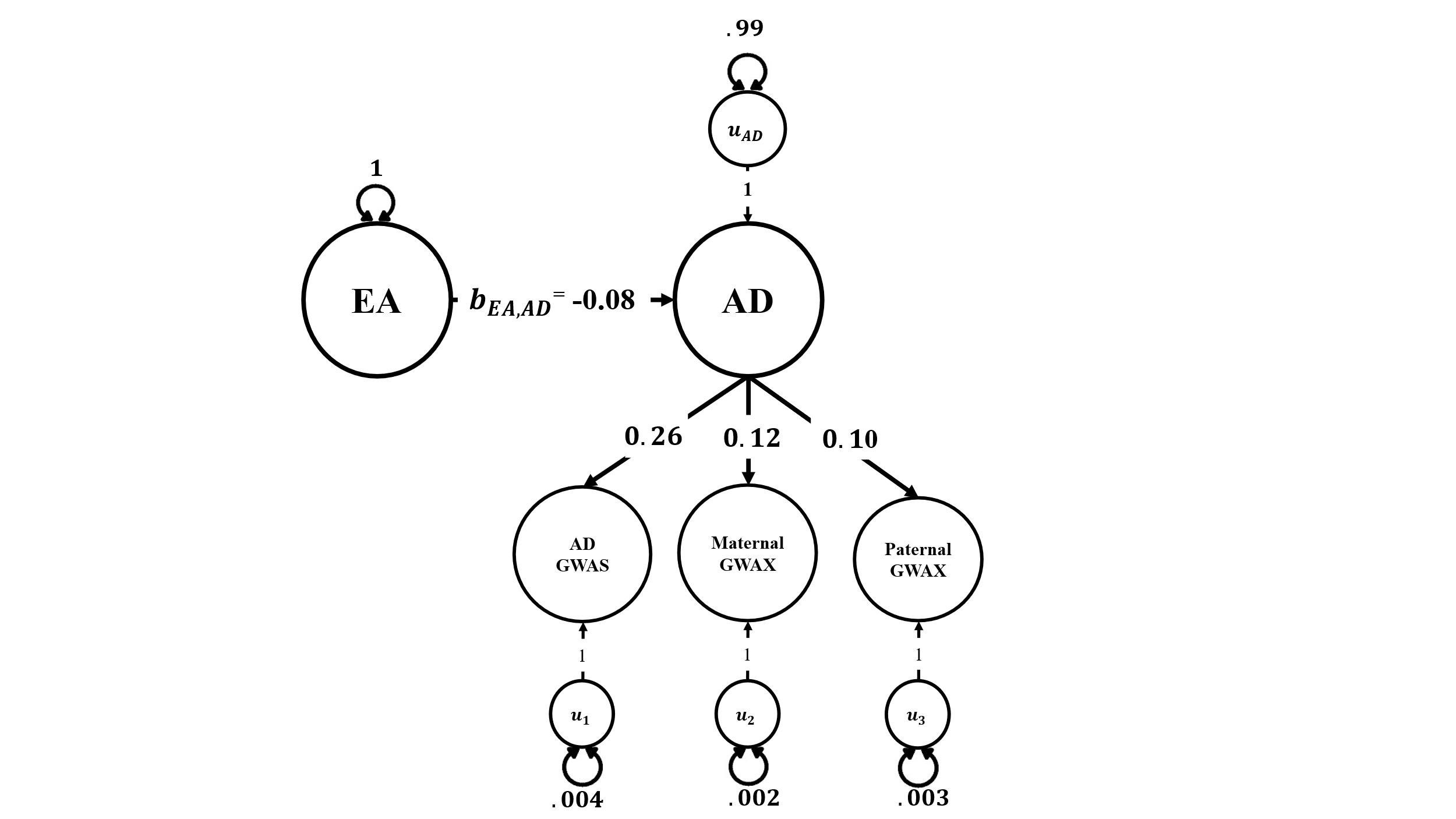
**Supplementary Figure S1.** Common pathway model for Alzheimer’s disease (AD) common factor regressed on educational attainment (EA)


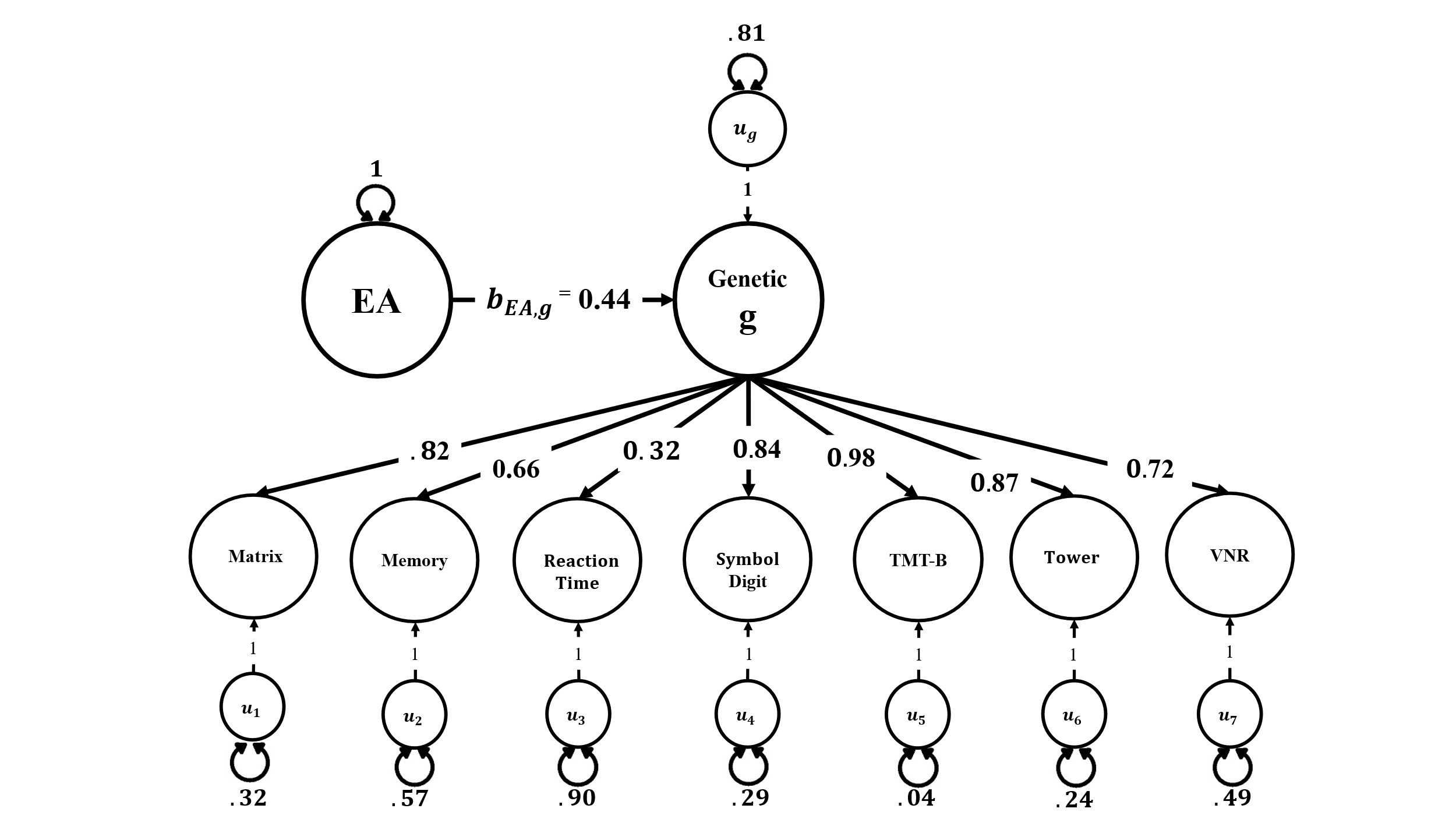
**Supplementary Figure S2.** Common pathway model for genetic g regressed on educational attainment (EA).


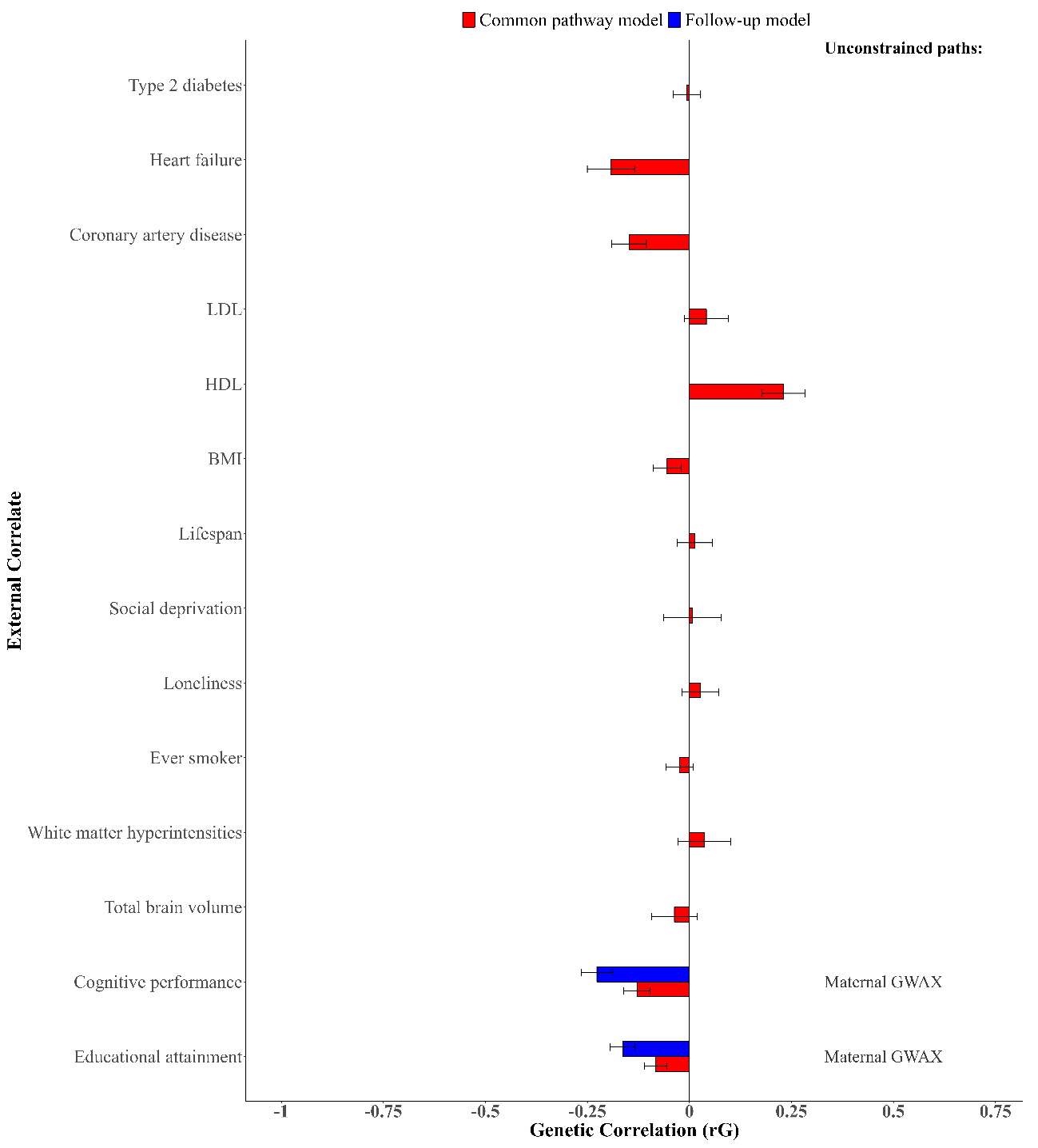


**Supplementary Figure S3.** Genetic correlations across external correlates and the Alzheimer’s Disease (AD) factor.
*The common pathway model assumes that all genetic associations between the external GWAS correlates and the individual indicator phenotypes are mediated through the common factor. The follow-up model corresponds with a model with unconstrained direct pathways to the outlier traits presenting significant deviations from the expectations under the common pathway model according to the three-test criteria of heterogeneity (see Methods)*


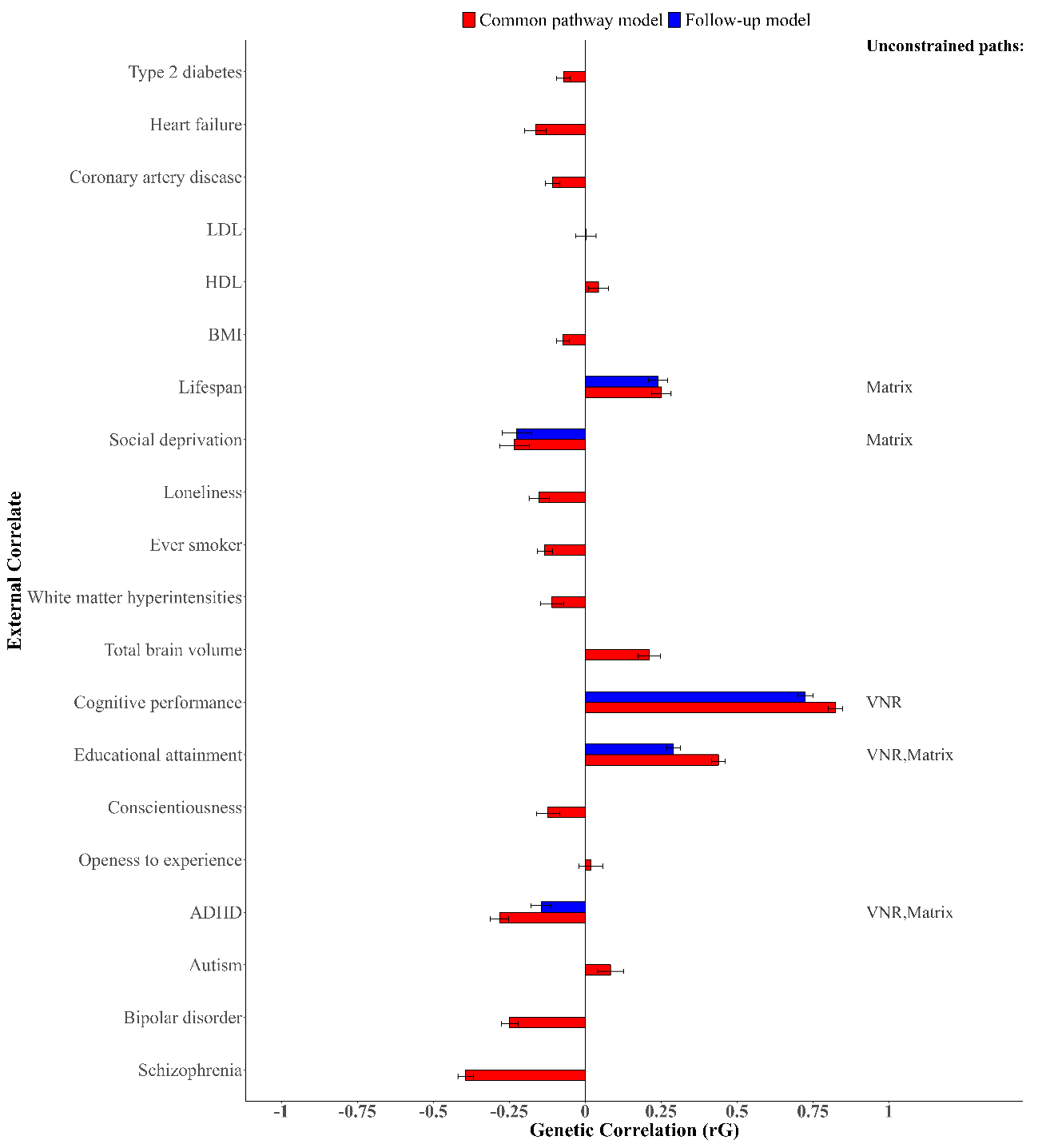


**Supplementary Figure S4.** Genetic correlations across external correlates and the genetic *g* factor.
*The common pathway model assumes that all genetic associations between the external GWAS correlates and the individual indicator phenotypes are mediated through the common factor. The follow-up model corresponds with a model with unconstrained direct pathways to the outlier traits presenting significant deviations from the expectations under the common pathway model according to the three-test criteria of heterogeneity (see Methods).*


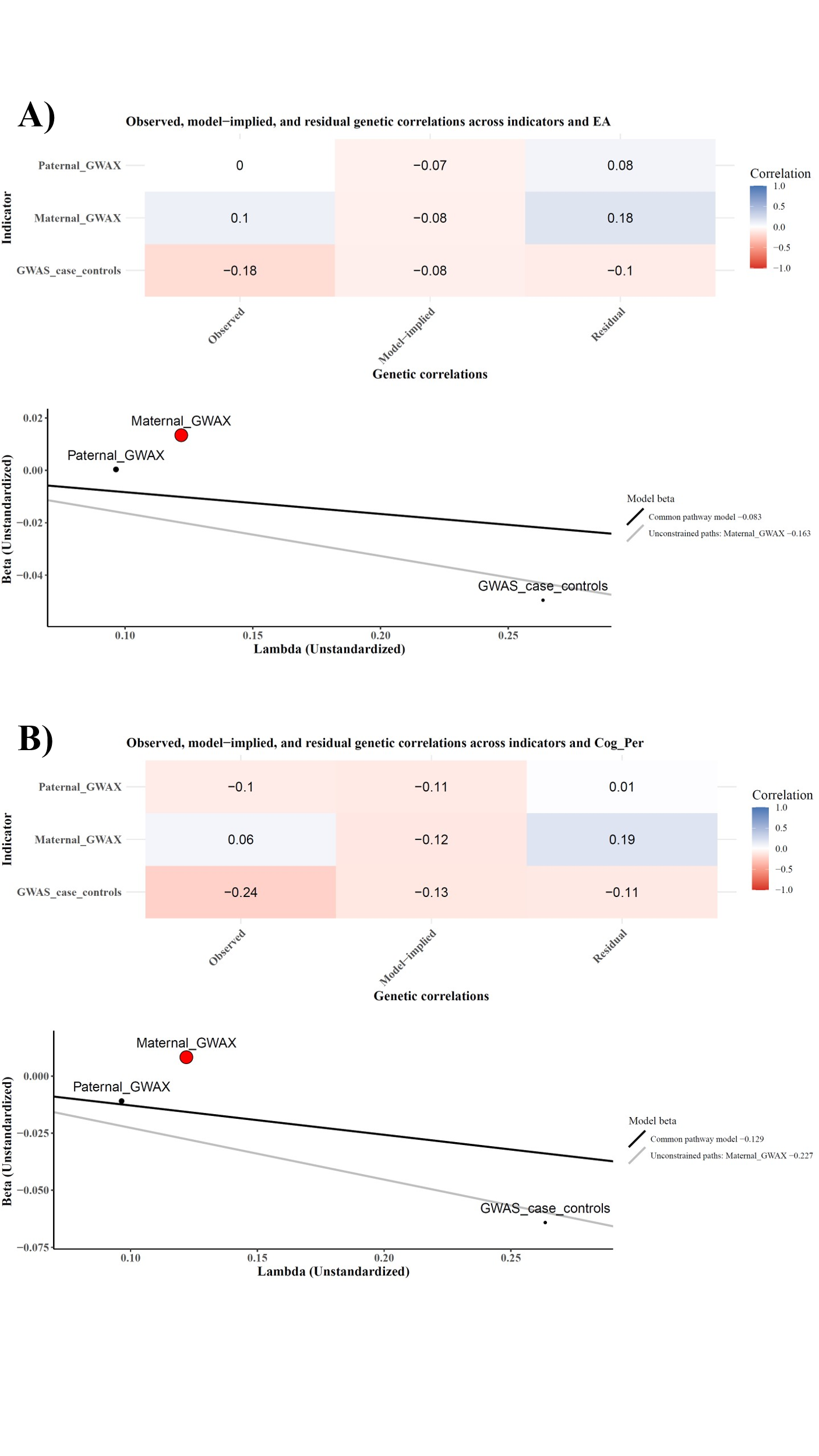


**Supplementary Figure S3.** Observed, model-implied, and residual genetic correlations between the three AD indicators (Paternal GWAX, Maternal GWAX, and case-control GWAS of Alzheimer’s disease), educational Attainment (**A**) and cognitive performance (**B**). Scatterplot of unstandardized beta coefficients of EA (**A**) and cognitive performance (**B**) on the three AD indicators against the unstandardized factor loadings of the three indicators on the AD factor described in de la Fuente et al. (2021). The size of the dots corresponds to the inverse of the variance of the unstandardized beta coefficient.

**
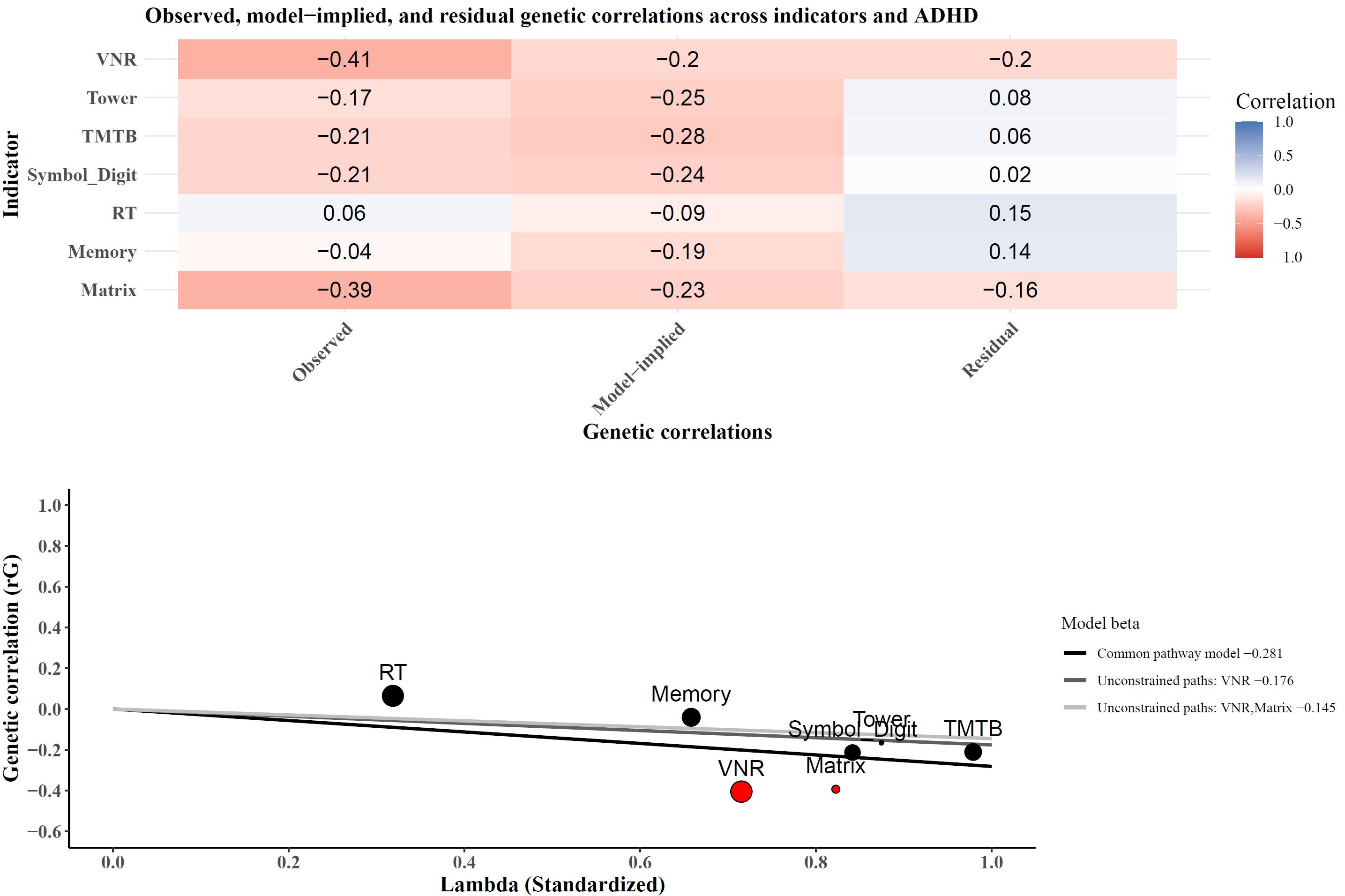
**

**Supplementary Figure S4.** Observed, model-implied, and residual genetic correlations between the seven indicators of the genetic *g* factor and ADHD (top). Scatterplot of unstandardized beta coefficients of ADHD on the seven indicators of the genetic *g* factor against the unstandardized factor loadings of these on the genetic g factor (bottom). The size of the dots corresponds to the inverse of the variance of the unstandardized beta coefficient.


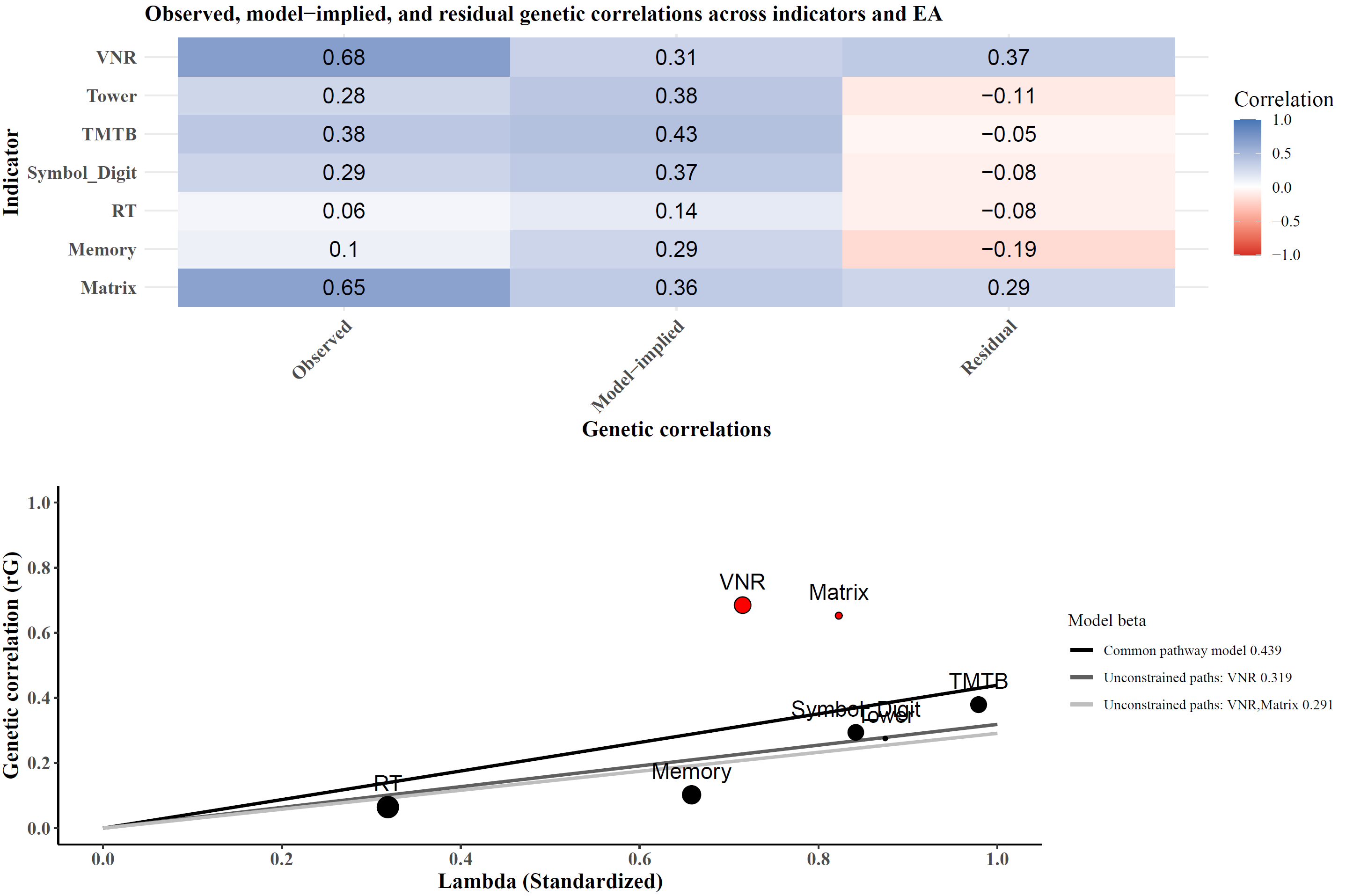


**Supplementary Figure S5.** Observed, model-implied, and residual genetic correlations between the seven indicators of the genetic *g* factor and educational attainment (top). Scatterplot of unstandardized beta coefficients of educational attainment on the seven indicators of the genetic *g* factor against the unstandardized factor loadings of these on the genetic g factor (bottom). The size of the dots corresponds to the inverse of the variance of the unstandardized beta coefficient.

**
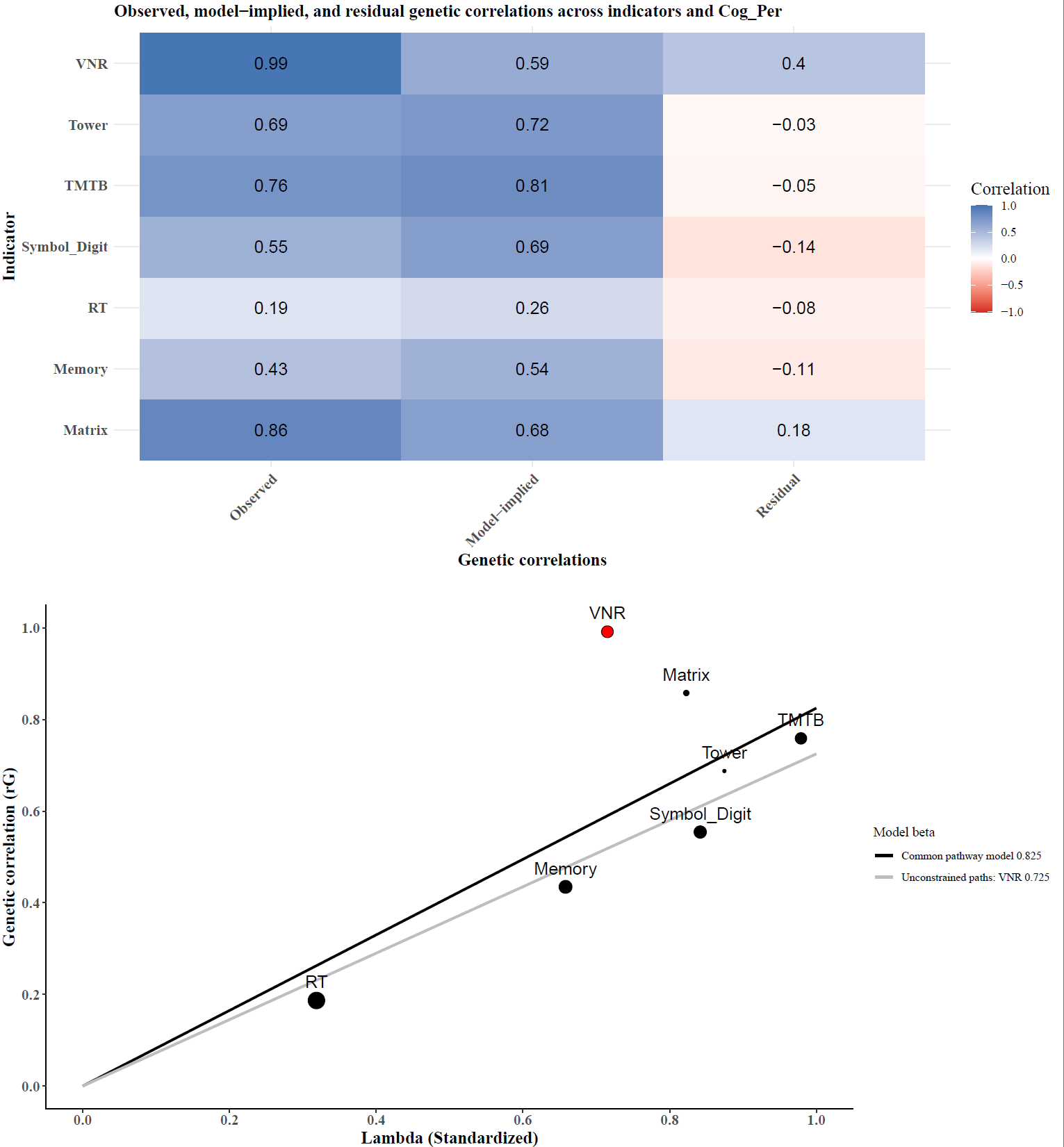
**

**Supplementary Figure S6.** Observed, model-implied, and residual genetic correlations between the seven indicators of the genetic *g* factor and a separate GWAS of cognitive performance (top). Scatterplot of unstandardized beta coefficients of cognitive performance on the seven indicators of the genetic *g* factor against the unstandardized factor loadings of these on the genetic g factor (bottom). The size of the dots corresponds to the inverse of the variance of the unstandardized beta coefficient.

**
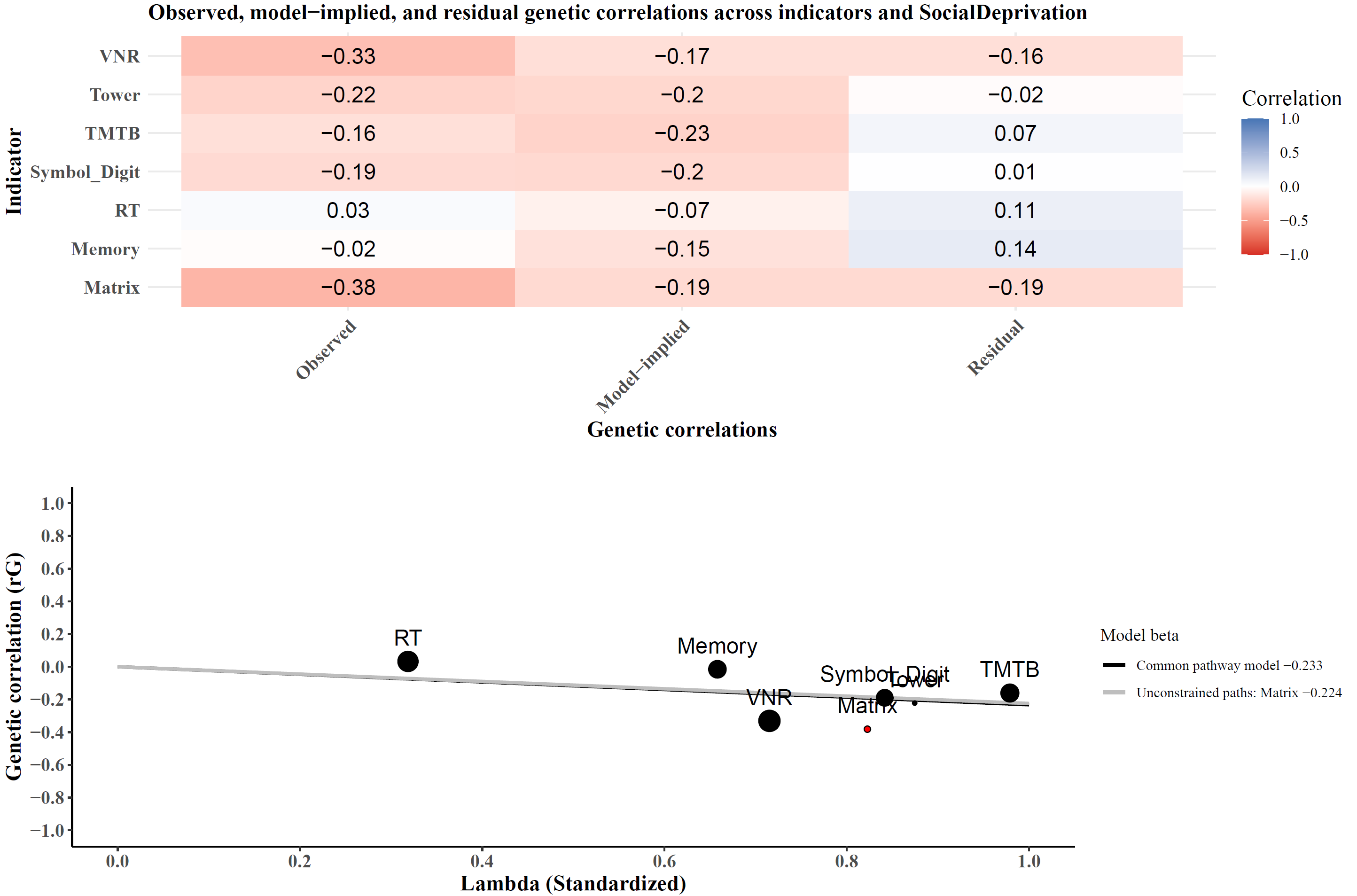
Supplementary Figure S7.** Observed, model-implied, and residual genetic correlations between the seven indicators of the genetic *g* factor and social deprivation (top). Scatterplot of unstandardized beta coefficients of social deprivation on the seven indicators of the genetic *g* factor against the unstandardized factor loadings of these on the genetic g factor (bottom). The size of the dots corresponds to the inverse of the variance of the unstandardized beta coefficient.

**
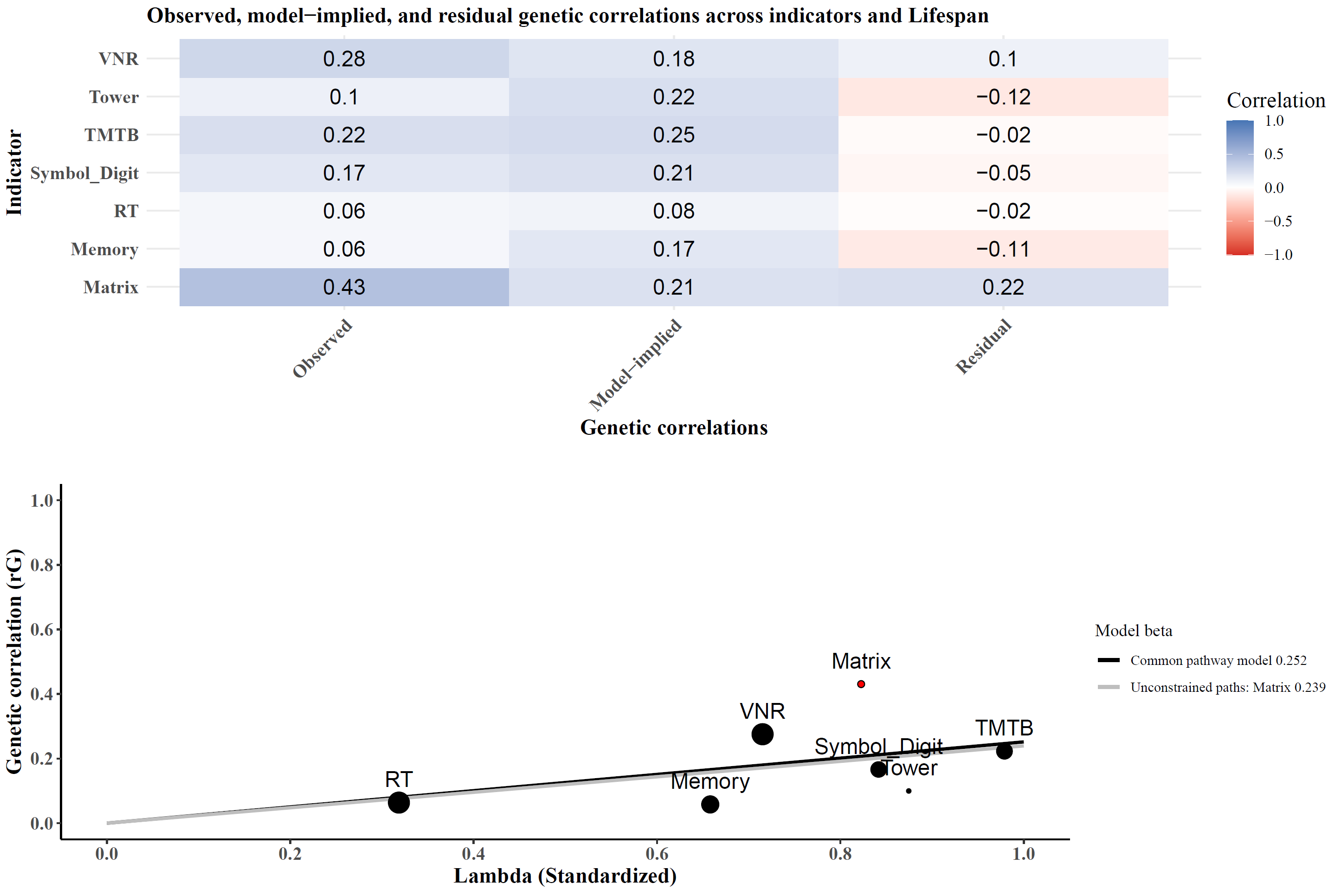
**

**Supplementary Figure S8.** Observed, model-implied, and residual genetic correlations between the seven indicators of the genetic *g* factor and lifespan (top). Scatterplot of unstandardized beta coefficients of lifespan on the seven indicators of the genetic *g* factor against the unstandardized factor loadings of these on the genetic g factor (bottom). The size of the dots corresponds to the inverse of the variance of the unstandardized beta coefficient.
